# pH variation impacts molecular pathways associated with somatic cell reprogramming and differentiation of pluripotent stem cells

**DOI:** 10.1002/rmb2.12346

**Published:** 2020-09-13

**Authors:** Narae Kim

**Affiliations:** ^1^ Nucleic Acid Chemistry and Engineering Okinawa Institute of Science and Technology Graduate University Okinawa Japan

**Keywords:** alternative splicing, cell differentiation, culture media, induced pluripotent stem cells, mitochondria

## Abstract

**Rationale:**

The study of somatic cell reprogramming and cell differentiation is essential for the application of recent techniques in regenerative medicine. It is, specifically, necessary to determine the appropriate conditions required for the induction of reprogramming and cell differentiation.

**Methods:**

Based on a comprehensive literature review, the effects of pH fluctuation on alternative splicing, mitochondria, plasma membrane, and phase separation, in several cell types are discussed. Additionally, the associated molecular pathways important for the induction of differentiation and reprogramming are reviewed.

**Results:**

While cells change their state, several factors such as cytokines and physical parameters affect cellular reprogramming and differentiation. As the extracellular and intracellular pH affects biophysical phenomena in a cell, the effects of pH fluctuation can ultimately decide the cell fate through molecular pathways. Though few studies have reported on the direct effects of culture pH on cell state, there is substantial information on the pathways related to stem cell differentiation and somatic cell reprogramming that can be stimulated by environmental pH.

**Conclusion:**

Environmental pH fluctuations may decide cell fate through the molecular pathways associated with somatic cell reprogramming and cell differentiation.

## 1 INTRODUCTION

In the context of early embryonic development in vertebrates, the cytokines secreted from the environment along with the signals within the differentiating cells stimulate the downstream molecular pathways that decide cell fate.^1^, ^2^ These processes are controlled by complex relationships, including those between signaling pathways and transcription factors which lead to the differentiation of specific cell lineages.^3^, ^4^ Simultaneously, the cells are exposed to rapid changes in the physical environmental factors such as temperature, pH, and osmotic pressure. These external stressors stimulate signaling pathways in cells to control cell behavior.^5^ Specific pathways activated by physical changes in cells play important roles in cell differentiation and pluripotency. For instance, a drastic increase in environmental temperature can induce the activation of heat‐shock proteins (HSP). HSP90, specifically, functions as a chaperone, while the HSP90β isoform is reportedly required for placental labyrinth development.^6^ Also, the lack of Hsp90α compromises sperm production.^7^ Furthermore, temperature stress alters the regulatory region of genes associated with pluripotency in human embryonic stem cells (ESCs).^8^ Hence, the manner in which cells respond to environmental changes can effectively regulate how they alter their state. However, the mechanisms that control such processes are not yet fully understood.

Nevertheless, pH may be one such factor that impacts cell fate. pH fluctuation serves as environmental stress that induces several responses in cells, resulting in the phenotypical change of cell behavior. Although a few studies have reported on the extracellular and intracellular impact imposed by pH on cell differentiation, the precise molecular pathway has not been fully explored.^9^ Hence, investigating the molecular phenomena related to pH fluctuation is necessary to elucidate the pH‐mediated regulation of cell fate. However, considering that environmental pH fluctuations are accompanied by a wide range of biological phenomena, as molecular pathways are interconnected, the role of pH may be ambiguous. Therefore, the phenomena that are a direct result of pH variation must be investigated with respect to similar phenomena and molecular pathways strongly associated with pluripotency and differentiation.

**FIGURE 1:**
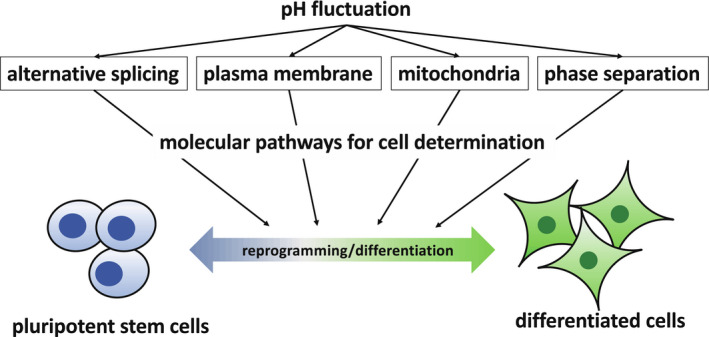
A scheme of the outline of this review

To observe the effects of pH on cell fate direction in vitro, the differentiation of pluripotent stem cells (PSCs) and somatic cell reprogramming account for the most drastic changes in cell status. Though the study of PSCs, including ESCs, is widely applied in regenerative medicine and for understanding embryonic development, the molecular pathways associated with pH fluctuations are not completely understood. In our previous paper, in addition to cell differentiation, the effects of pH on differences in colony formation and timing of somatic cell reprogramming were observed; thus, pH may affect the direction of cell differentiation and somatic cell reprogramming.^5^ Therefore, this review focuses on promising molecular pathways that support this hypothesis (Figure 1).

## 2 STATE OF PLURIPOTENT STEM CELLS

Pluripotency refers to the ability of a cell to differentiate into multiple cell types. Specifically, once an egg becomes fertilized, the individual organism achieves increasing complexity as development proceeds following exposure to a number of factors. During this process, the cells differentiate into other cell types and ultimately into somatic cells, which effectively lose their ability to differentiate.

Embryonic stem cells are one such pluripotent cell type derived from the inner cell mass (ICM) of a vertebrate blastocyst, the pluripotency and self‐renewal capacity for which can be maintained in vitro under appropriate conditions.^10^ In the case of mice, ESCs can be maintained with leukemia inhibitory factor (LIF) and serum. Mouse ESCs differentiate into three germ layers and also contribute to the chimera and germ cells. Meanwhile, epiblast stem cells (EpiSCs) are derived from post‐implantation embryos with rather limited differentiation capacity compared to ESCs and do not significantly contribute to chimera.^11^ The PSC state of the former is called a naïve state, and the latter is called a primed state.^11^ Mouse ESCs are in a naïve state that can be supported via inhibition of specific signaling pathways, including GSK3β and MEK, in addition to LIF.^12^ Meanwhile, EpiSCs are defined as having primed pluripotency, which can be maintained in culture via the application of specific cytokines, such as activin A and basic fibroblast growth factor (bFGF).^11^ Furthermore, the naïve ESC colonies are morphologically domed with a rounded shape, while that of primed EpiSCs is mono‐layered and flattened. Thus, there are distinct states of pluripotency. In addition to these naïve and primed states, a recent study hypothesized the existence of formative pluripotent cells occurring as an intermediate stage during the induction of EpiSC‐like cells from naïve ESCs, in which cells have the potential to differentiate into germ cells.^2^, ^13^

The characteristics of human ESCs are more similar to mouse EpiSCs.^14^ Hence, the conversion of human ESCs to a naïve state has been investigated to extend their utilities in areas of reproductive medicine such as germ cell induction.^15^ Specifically, altering media conditions, as well as overexpressing specific exogenous genes, have been implemented as primary approaches that have led to success in this research area.^16^, ^17^ Similarly, naïve ESCs from other vertebrates have been characterized in relation to mouse ESCs.^17^, ^18^, ^19^

Induced pluripotent stem cells (iPSCs), derived from somatic cells via overexpression of several transcription factors, have similar characteristics as those of ESCs. Since the iPSCs have first reported in 2006, which derived from mouse fibroblasts by co‐overexpression of OCT4, SOX2, KLF4, and c‐MYC,^20^ and then human iPSCs in 2007.^21^ Considering that this strategy does not require sacrificing animal embryos, the generation of iPSCs has offered the incredible potential for advancements in the field of regenerative medicine and reproductive biology both in humans and in other vertebrates.^22^, ^23^ A wide range of studies, therefore, rely on the capacity of PSCs to differentiate into several cell types, both in vivo and in vitro.

Somatic cell reprogramming, for instance, can be affected, with respect to its reprogramming efficiency, by the pH of the culture medium.^5^ Although the generation and the application of iPSCs have been systematically improved, the effects of pH in the culture have not been fully explored, which may affect the state of pluripotency in iPSCs.^2^ Depending on the desired cell or organ, PSCs must be induced and maintained in appropriate conditions to maintain a pluripotent state.

## 3 PERTURBATION OF ALTERNATIVE SPLICING BY pH

In the nucleus, transcribed mRNA is subject to several modifications and is transferred to the cytoplasm. Alternative splicing (AS) is the process of cutting pre‐mRNA at splice sites and re‐organizing the RNA fragments to generate various mRNAs. Several mRNA splice variants are created using AS, which these variants produce protein isoforms depending on the different splicing patterns.

Fluctuations in extracellular pH can affect mRNA splicing. Tenascin‐C (TNC), an extracellular matrix (ECM) protein that includes fibronectin type III repeats, is a well‐studied example, and has several isoforms generated via AS. The short and long AS isoforms exhibit different adhesion activities in the environment.^24^, ^25^ Such AS variants were found to be dependent on the pH of the medium.^26^ TNC has been studied for its effects on tumorigenesis and embryogenesis.^24^, ^27^ Its splice variants are expressed in a broad range of cells and tissue, and the specific splicing patterns were found to change depending on the developmental stages.^28^

In terms of the control of AS, splicing factors (SFs) regulate AS and are known to directly interact with mRNA. Several SFs have been identified to date, one of which is SRSF6 that controls the AS of TNC.^28^, ^29^ SFs exhibit different behavior in response to stresses.^29^ For instance, the secondary structure of mRNA can become altered in vivo and in vitro.^30^ pH variation in the cell may control the binding affinity of SFs by RNA folding; however, this requires further investigation.

Particular splice variants have differential roles in pluripotent cells and differentiated cells. FOXP1 is one such transcription factor that has different splice variants in ES and somatic cells. Each isoform has a specific transcriptional role, binding to distinct positions in the genome in the respective cell types.^31^ Several pluripotency‐related proteins such as IGF‐1, OCT4, and FGF4, which are important to maintain pluripotency and direct cell fate, have AS isoforms.^32^, ^33^, ^34^ For instance, E‐cadherin, one of the hallmarks of PSCs, is a critical factor for cell‐cell adhesion and is an essential protein for maintaining a LIF‐dependent naïve state in ESCs. Its expression becomes decreased while N‐cadherin is increased as the differentiation of EpiSCs progresses.^32^, ^35^, ^36^ Moreover, the loss of endogenous E‐cadherin reduces the efficiency of reprogramming upon iPSC establishment.^36^ Though the presence of E‐cadherin splice variants has been also reported,^37^ as well as the other pluripotency‐related protein isoforms, little is known regarding the differential role of each of the resulting isoforms in cell differentiation and reprogramming.

The currently available comprehensive NGS‐based assays have revealed differences between splice variants in pluripotent and differentiated cells.^38^ These assays may further uncover new RNA variants as well as their roles, which may reveal the pH‐sensitive characteristics of these variants.

## 4 MITOCHONDRIAL ACTIVITY IN STEM CELLS AND VARYING pH

One of the important pathways affected by pH is mitochondrial activity. Mitochondria are an essential organelle for ATP production via the membrane potential caused by the proton gradient. In PSCs, the metabolic changes in mitochondria are critical for pluripotency.^39^, ^40^ The metabolism in ESCs is dependent on anaerobic glycolysis carried out by the immature mitochondria with round morphology, while differentiated cells such as fibroblasts produce energy via aerobic respiration with a high‐density matrix.^41^, ^42^ The conversion of somatic cells to the pluripotent state follows changes in the mitochondrial metabolic status.

Hypoxic environments, which stimulate glycolysis, can support the maintenance of the pluripotency of ESCs.^41^, ^43^ The transcriptional activation of hypoxia‐inducible factor 1 (HIF1) has a central role in mitochondrial activity conversion. HIF1 stabilization, which plays an important role in the maintenance of naïve ESCs, can also be induced by acidic environmental pH.^44^ Stabilized HIF1 inhibits differentiation of ESCs and increases the efficiency of iPSC generation.^45^ Moreover, the direct inhibition of mitochondrial respiration can promote pluripotency.^46^ Thus, although the mitochondrial activity is influenced by the cytoplasm owing to the role of the proton gradient, it can also be affected by the extracellular environment due to the correlation between the external and internal pH.^47^ Therefore, the pH in culture may have a role for the cell state on somatic cell reprogramming and cell differentiation.

## 5 EFFECTS OF THE PROTON GRADIENT ON THE PLASMA MEMBRANE AND THE SIGNALING PATHWAYS

The sodium hydrogen exchanger 1 (NHE1), localized on the plasma membrane, exchanges the extracellular Na^+^ for intracellular H^+^ and, thus, has a pivotal role in the maintenance of intracellular proton homeostasis in cells.^48^, ^49^ Specifically, NHE1 activity is well studied in cancer in relation to environmental pH changes.^48^ Furthermore, the overexpression of NHE1 induces cell death in human iPSCs, however, not in differentiated cells such as mesendoderm‐like cells.^50^ The increase in intracellular pH as a result of NHE1 activity is required for early ESC differentiation,^51^ implying that the rise in intracellular pH may affect intracellular dynamics, thus inducing changes in cell fate.

**FIGURE 2:**
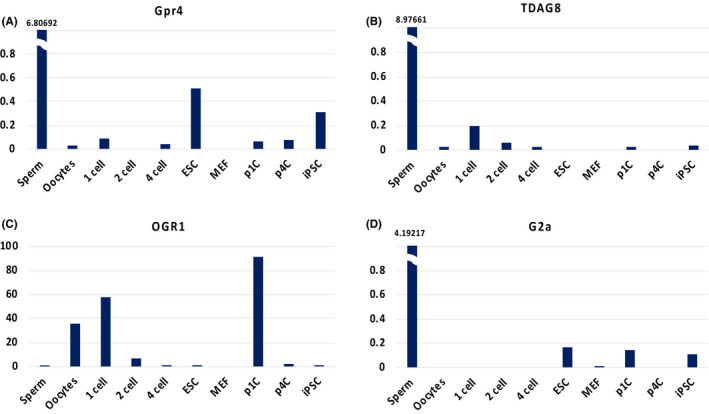
Transcriptome expressions of proton‐sensing GPCRs in each developmental stage according to DBTMEE database.^55^ iPSC, induced pluripotent stem cell; MEF, mouse embryonic fibroblast; p1C, parthenogenetic one‐cell; p4C, parthenogenetic four‐cell. The *y*‐axis indicates fragments per kilobase of transcript per million fragments mapped (FPKM).

As the pH varies with the proton gradient, direct cell surface receptors also play a critical role. The G‐protein coupled receptor (GPCR) family exists in the plasma membrane of various tissues. GPCRs sense external signals such as light, chemicals, and hormones. Proton‐sensing GPCRs, such as GPR4 (GPR84), OGR1 (GPR68), TDAG8 (GPR65), and G2A (GPR132), become activated approximately at pH 6.5.^52^, ^53^ Studies on cancer cells and immune cells suggest that certain GPCRs influence cell reprogramming^54^; however, only limited information is available regarding the role of proton‐sensing GPCRs in somatic cell reprogramming and cell differentiation. According to the database of mouse early embryos, for instance, the transcriptome of OGR1 is highly expressed in oocytes, the one‐cell stage, and parthenogenetic one‐cell stage (Figure 2).^55^ In contrast, TDAG8 is rarely expressed in embryonic fibroblasts and ESCs, but is expressed in sperm (Figure 2).^55^ Also, GPR4 and G2A expressions are observed in sperm and, to a lesser extent, in ESCs and iPSCs (Figure 2).^55^ Thus, these proton‐sensing GPCRs are differentially expressed in somatic cells and early stages of development. Furthermore, in the one‐cell to four‐cell stages, which have totipotent characteristics rather than pluripotency,^10^, ^56^ certain proton‐sensing GPCRs are expressed. The proton‐sensing GPCRs, therefore, may thus have distinct roles in each embryonic stage.

When the proton‐sensing GPCRs stimulate the signaling pathways, the signal reaches some molecules that are also related to somatic cell reprogramming and differentiation. For instance, the activation of the cyclic AMP/protein kinase A (PKA) pathway, which can be stimulated by TDAG8, has an inhibitory effect on somatic cell reprogramming.^52^ Moreover, the activation of PKA accelerates the early differentiation of ESC through epigenetic modifications of histone H3 lysine 9, which is one of the activity markers in the genomic region.^57^ Though there are some reports that proton‐sensing GPCRs in non‐pluripotent cells stimulate the signaling pathways that are important for somatic cell reprogramming and differentiation, the effects in ESCs and iPSCs remain unclear.

## 6 PHASE SEPARATION IN CELLS AND THE CONTROL OF DIFFERENTIATION

Recently, liquid‐liquid phase separation (LLPS) has been investigated on the study of cellular RNA. This phenomenon involves the formation of droplets in a cell including those enriched in mRNA, RNA‐binding proteins, and/or supporting molecules, providing active translation spots. For instance, stress granules, which include P body, germline P granule, and nucleoli, are phase‐separated droplets involved in several biological processes.^58^ The formation conditions for LLPS can be influenced by stress, including pH fluctuation.^58^, ^59^, ^60^

Another recent study demonstrated the pivotal role of LLPS in cell differentiation. During neural stem cell differentiation, in which the mitotic retention of transcription factors is important for the terminal neuronal differentiation, the phase‐separated droplets, including homeodomain transcription factor Prospero and heterochromatin protein 1 retained on chromatin, drive heterochromatin domain expansion, directing terminal neural differentiation.^61^

With regard to the formation of granules, poly(A)‐binding protein (Pab1) is one of the markers of stress granules.^62^, ^63^ In yeast, mRNA‐bound Pab1 is demixed by thermal and pH stresses. The demixed Pab1 is phase‐separated by self‐interacting into gels releasing mRNA, which eventually enter stress granules.^62^, ^63^ However, much of the actual function of phase separation activity, such as the formation of stress granules in mammalian cells, still remains to be elucidated.

Thus, differentiation can be affected via the formation of LLPS.^61^, ^62^, ^63^ Further investigations may reveal the existence of LLPS related cell differentiation which sensitive to pH, whereas advanced studies are required in mammalian cells.

## 7 CONCLUSION

**FIGURE 3:**
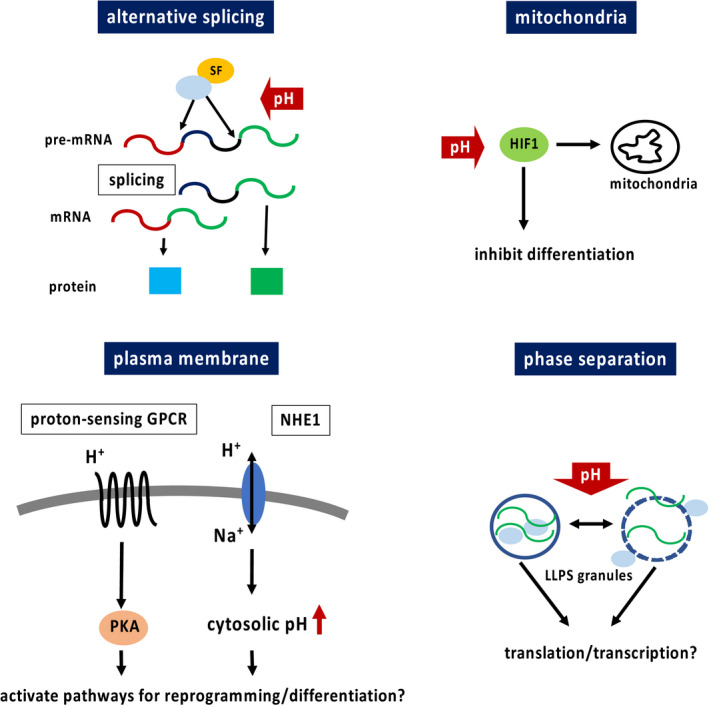
pH‐related phenomena discussed in this review. H^+^, proton; HIF1, hypoxia‐inducible factor 1; LLPS, liquid‐liquid phase separation; Na^+^, sodium ion; NHE1, sodium hydrogen exchanger 1; PKA, protein kinase A; SF, splicing factor

The present review particularly focused on AS, mitochondria, plasma membrane, and phase separation, which are known to have a strong association with somatic cell reprogramming and cell differentiation. Narrowing the field of view on each phenomenon reveals that each of these factors impacts cell behavior through environmental pH‐mediated direct or indirect activation of signaling pathways or via physical impact. Although there are a few reports on the direct influence of pH on cell fate, pH fluctuation can also have comprehensive effects. Moreover, phase separation is a relatively recent field of study; therefore, the physical dynamics may implicate numerous unknown effects on cell fate. The pH‐related phenomena discussed in this review are summarized in Figure 3.

Understanding the ideal conditions for cell differentiation and somatic cell reprogramming is necessary for the study of regenerative medicine. Fluctuation in the physical surroundings of cells can be instrumental in deciding their fate, and the engagement of cell behavior and downstream pathways is necessary to improve the probability of appropriate cell fate direction. Understanding the precise conditions and complex signaling pathways is required for recent industrial large‐scale culture and 3D culture for organoid research, respectively. Moreover, organizing these phenomena based on molecular approaches and adjusting the cell culture conditions may serve to further optimize these applications.

## DISCLOSURES

*Conflict of interest:* Narae Kim declares that she has no conflict of interest. *Human/animal rights:* This article does not contain any studies with human or animal subjects performed by the author.
